# Diaphragm’s Role as a Systems-Connector Muscle: A Narrative Review

**DOI:** 10.7759/cureus.94679

**Published:** 2025-10-15

**Authors:** Bruno Bordoni, Bruno Morabito, Allan R Escher

**Affiliations:** 1 Physical Medicine and Rehabilitation, Foundation Don Carlo Gnocchi, Milan, ITA; 2 Physical Medicine and Rehabilitation, School of Osteopathic Centre for Research and Studies, Milan, ITA; 3 Oncologic Sciences, University of South Florida Morsani College of Medicine, Tampa, USA; 4 Anesthesiology/Pain Medicine, H. Lee Moffitt Cancer Center and Research Institute, Tampa, USA

**Keywords:** chf, chronic heart failure, chronic obstructive pulmonary disease, copd, diaphragm, glymphatic system, inflammation, lymphatic system, osteopathic treatment

## Abstract

The diaphragm muscle (DM) is the most complex muscle in the entire musculoskeletal system, and it is associated with the most systemic actions. It is often labeled a pump, reflecting its role in the respiratory and circulatory systems. In reality, the DM's functions are broader, and the term "pump" does not adequately capture its importance. Clinically, DM is rarely considered to improve movement, cognitive and emotional functioning, or its influence on the immune response. Clinically and based on two-dimensional instrumental examinations, it is believed that DM "flattens" during inspiration or in the presence of chronic obstructive pulmonary disease (COPD). In contrast, we highlight, based on literature employing three-dimensional clinical instrumentation, that DM does not change its morphology during inflation. Therefore, the importance of DM functional assessment lies not in its shape but in its ability to move. To conclude, we propose a hypothesis for an adjective that best reflects the systemic functional complexity of DM.

## Introduction and background

The diaphragm muscle (DM) is the main player in the mechanisms that allow the lungs to function correctly, allowing adequate blood flow to the lung structures and creating the pressures essential for the movement of air through the respiratory system [1,2]. Without sufficient DM strength, the lungs would develop functional alterations, resulting in local and systemic pathologies [3-6]. DM alterations are not necessarily attributable to chronic pathologies, such as chronic obstructive pulmonary disease (COPD) or chronic heart failure (CHF), where, for reasons that are not entirely clear, the DM undergoes profound pathological changes, at the macroscopic and ultrastructural level [2,6].

Changes in posture and other biomechanical motivations (increased activity of respiratory accessory muscles) can induce adaptations to the rib cage (reduced rib cage expansion), to the abdominal area (reduced abdominal excursion), which hinder the ability of the DM to contract and relax exhaustively, both in conditions of overt disease and in healthy people [7]. A recent study has shown that a population of healthy and athletic subjects (n=1933) showed an inadequate use of DM in the breathing pattern in over 90% [8]. Dysfunctional breathing occurs when the physiological breathing pattern is absent; a functional breathing pattern involves the movement of the diaphragm and intercostal muscles, and allows for influence on the movement of the rib cage and abdomen [8]. Another study has shown an incorrect intervention of the DM in healthy and elite athletic subjects, where over half of the people did not breathe in an optimal way [9]. A physiological breathing scheme enables us to use the neurological and biomechanical mechanisms involved in breathing in an exhaustive manner.

Correct DM movement (“proper breathing”) leads to better results in spirometric tests [9]. Other DM dysfunctions can occur due to disorders that are not strictly pulmonary, such as the presence of postural alterations and antalgic habits due to lumbar and cervical pain, temporomandibular joint pain, and neuromotor alterations (e.g., scapular dyskinesis), chronic ankle instability [10,11]. Furthermore, depending on the position of the person undergoing a spirometric test, the values ​​deduced by the clinician can change, just as lung volumes do not describe the behavior and the real extent of intervention of the diaphragm in the respiratory act [10,7]. In clinical practice and in literature, ultrasound is the most commonly used tool to verify the movement of DM; other tools employed, depending on the capacity of the healthcare facility and/or the motivation for the investigation, are chest fluoroscopy, plethysmography, functional thoracic MRI, electromyography, and CT [12-15]. Other investigation strategies are spirometry, maximum inspiratory pressure (PImax), and sniff nasal inspiratory pressure (SNIP), but these do not directly measure and/or isolate the function of the DM with respect to other muscles involved in respiration (accessories) [15].

In the literature, DM is compared to a piston or a pump due to its ability to decrease pleural pressures during inspiration by varying the pressure gradients between the alveoli and allowing gas exchange [16,17]. According to some studies, the contractile capacity of the diaphragm is correctly matched with the inflow and outflow of air into and from the respiratory tract, through considerations based on studies with two-dimensional instruments or focusing on the intrinsic structure of the DM. These visions do not allow for a three-dimensional view, nor even a view inside a body system.

Furthermore, when the DM contracts during inflation or undergoes a chronic inspiratory posture, it is defined as a "flattened" state [7,18]. Considering DM only as a "pump" fails to include other, more complex functions related to breathing; furthermore, the terminology (pump) does not reflect the fact that the displaced fluids and the inhaled and exhaled air undergo tortuous, non-linear movements. Also, according to the data we have available and which will be cited in the following paragraphs, the term defining DM as flattened is derived from two-dimensional, not three-dimensional, instruments, which demonstrate that the morphology of the DM does not change during breathing.

In this narrative review, we searched the PubMed databases for the relevant literature. The search covered the period from 1990 to 2025. No specific limits were put forward in the study selection criteria. The search strategy included a combination of the following search terms: diaphragm movement, zone of apposition (ZOA), diaphragm x-ray, diaphragm magnetic resonance imaging, diaphragm computed axial tomography, diaphragm kinetics, diaphragm kinetics three-dimensional instrumental examinations, and diaphragmatic pump. Finally, the reference lists in the retrieved articles were examined to identify any additional relevant articles. The article reviews how the diaphragm moves, its functions, and briefly its anatomy. This narrative review aims to challenge the idea of considering DM as a pump or defining it as flattened. Finally, we suggest a new adjective that better captures the complexity of diaphragmatic movement.

## Review

Anatomical notes

We can distinguish three anatomical areas where DM is involved. The sternal area, where muscle bundles attach posteriorly to the xiphoid process, whose fibers derive from the rib area; the sternal diaphragmatic fibers form the Larrey's fissure or sternocostal triangle [19]. Larrey’s fissure is crossed by internal thoracic and superior epigastric vessels [19].

The rib area, where the DM attaches to the posterior and superior portion of the last six ribs laterally, interdigitating with the transversus abdominis muscle [20]. The vertebral and costal area, where the DM involves the anterior and lateral portion of the dorsal vertebrae (11-12), up to touching the 4th lumbar vertebra. The pillars are divided into medial and lateral. The right medial pillar originates from the dorsal area anteriorly (T11), up to the fourth lumbar vertebra (L4); the left medial pillar, smaller and thinner, always originates from T11, up to L3 [20]. The two pillars cross in their path, forming the esophageal hiatus and the aortic hiatus (or median arcuate ligament), at the level of T11-T12. At the level of the esophageal hiatus, we find two diaphragmatic muscle strips. From the right medial pillar, superiorly and posteriorly, arises the Low's muscle, which fuses with the left pillar [21].

Posterior to the Low's muscle and joining the right and left pillars, we find the transversus intertendinous muscle [21]. Below the medial pillar, there is another muscle, the Hilfsmuskel muscle, also originating from the DM, which touches the celiac artery and fuses with the Treitz muscle or suspensory duodenum [21]. The hiatus of the vein is found on the right and posteriorly, at the level of T10-T11 [22].

The remaining diaphragmatic body involves the lateral pillars, which form the intermediate ligaments (spinous process and transverse process of L1), merging above the psoas muscle, and the lateral ligaments (transverse process of L1 and apex of the last rib), merging with the quadratus lumborum muscle [20]. The diaphragmatic portion containing a greater quantity of protein (number of fibres) and with a longitudinal orientation is the lateral-posterior one, while the remaining lateral-anterior area is poor in sarcomeres and has more oblique vectors [22].

The DM has a surface area of ​​approximately 900 square cm, which is innervated by the phrenic nerve (C3-C5), and only a small portion of the muscle is innervated by the vagus nerve (cranial nerve X) [20,23]. The vagus nerve innervates the Low's muscle and the transversus intertendinous muscle, i.e., the diaphragmatic crural muscle area that is involved in controlling the passage of the bolus towards the stomach [21,24]. DM is highly vascularized through a complex network arising from the intercostal and internal thoracic phrenic arteries [25]. DM is a muscular structure rich in lymphatic vessels, which are connected to the Chyli cistern (subdiaphragmatic area) and the peritracheobronchial lymph nodes (supradiaphragmatic area) [26,27].

Movement of the diaphragm

**Figure 1:**
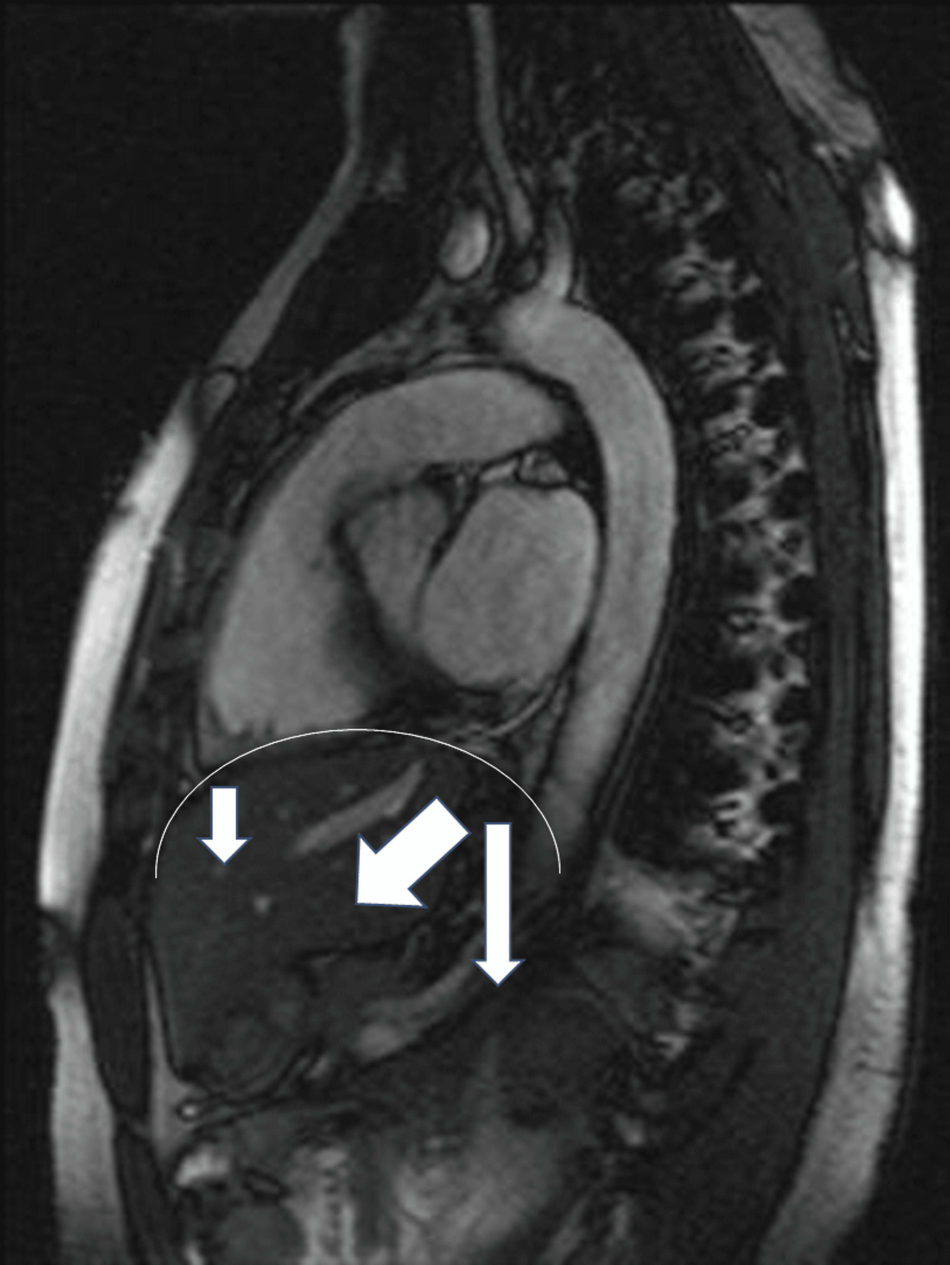
An illustration of how the diaphragm moves during inspiration, with caudal and anterior vectors, with an overall oblique direction* ^*^[4]

The ZOA of the diaphragm is the portion in contact with the internal area of the rib cage (diaphragmatic epimysium and endothoracic fascia), and represents approximately 60% of the entire surface of the DM [22]. When DM contracts, it can exploit this area to increase its contraction efficiency, pushing the ribs laterally during inspiration and with less emphasis antero-posteriorly [22]. During a eupneic act, DM can perform a range of motion of approximately 0.9-2 cm, while with a forced breath, it can perform a movement of approximately 10 cm [22]. During inspiration, DM is confronted with abdominal (PA) and pelvic pressure, which must allow an adequate diaphragmatic contraction [22]. ZOA conditions the movement of DM with a caudal vector when there is inflation, while PA will facilitate an anterior-caudal movement [28]. Adding the two vectors, DM has an oblique direction, where approximately 40% of the movement is performed by the lateral-posterior portion (Figure 1) [22,29].

The right hemidiaphragm is higher than the left side due to the presence of the liver by about 1.9 cm, which difference decreases slightly during the end of inspiration, with an increasingly cephalad position of about 1.5 cm [28]. To compensate for this positional difference, the right phrenic nerve is shorter than the left one; the right electrical impulse arrives faster than the slightly slower left electrical impulse. The right side moves faster than the left side. This additional difference allows for a nearly homogeneous speed of diaphragmatic movement [4].

With complete inspiration, the thickness of the DM is approximately 0.22 cm, while at the end of a complete expiration, the thickness is approximately 0.20 cm [4]. In the supine/prone position, the DM moves with a wider range, while in the upright position, it moves with less emphasis. This is because the DM is contracted in the upright position for its postural and movement functions, while in the supine position, it is more involved in respiration [6,22]. When the DM shortens during inspiration, its morphology does not change; it does not flatten, as demonstrated by studies [28,30]. Despite its limited thickness (2-3 mm), the DM is involved in 60-80% of the necessary ventilation [31].

When describing the movement of the diaphragm, we must remember that it is not a two-dimensional structure, as we see from the x-ray, MRI, and computed axial tomography. The most recommended examination to visualize the kinetics of the DM is two-dimensional (2D) or one-dimensional (M-mode) ultrasound, with greater ease in visualizing the right side, compared to the difficulty in visualizing the left hemidiaphragm (presence of the splenic flexure) [28]. As with any two-dimensional examination, the evaluation of the shape depends on the viewing angle and does not provide an accurate visualization of the morphology [28,32]. Only with three-dimensional instrumental examinations, such as three-dimensional computed tomography (3D-CT), is it possible to have a more correct evaluation [33].

There may be an inspiratory attitude of the DM as in COPD, where it appears flattened, but in reality, it is lower [34]. The thickness of the DM in chronic respiratory diseases is smaller, with a smaller ZOA surface area; a smaller thickness means less contraction and therefore less movement, and the three-dimensional morphology remains the same [28,30,34].

A common adjective for the diaphragm

Why do we commonly refer to the diaphragm muscle as a "pump"?

The term "pump" refers to a rhythmic movement of skeletal muscles, facilitating venous and lymphatic return, and indicates the ability of the skeletal muscles responsible for respiration to facilitate the flow of air into and out of the chest [35,36].

The skeletal muscles of the limbs, with their contraction during movement, or with the tension recorded at rest, create pressure gradients at the level of the superficial and deep venous system capable of pushing the venous blood in an anterograde direction [37]. In reality, a more recent study has challenged this hypothesis, demonstrating the absence of this anterograde movement [38]. Not all skeletal muscles act on the venous system in the same way. During walking, the superficial venous blood penetrates the gastrocnemius muscle through the perforating veins (retrogradely in the support and push phases), while the direction of the venous flow was anterograde with the contraction of the tibialis anterior and the lower tension of the gastrocnemius in the swing phase [38]. This mechanism allows for the creation of balanced pressures with respect to arterial pressures and counteracts the onset of venous hypertension. The venous flow does not follow a linear direction.

When we are upright, venous flow tends to flow toward the periphery, such as the pelvic area and lower limbs, due to gravity and the collapsibility of the veins. This lower venous accumulation is counteracted by the sympathetic nervous system (and a simultaneous reduction in baroreceptor activity) for a short period of time, attempting to prevent such accumulation and adequately restore central venous return [39]. If these mechanisms are not congruent, orthostatic hypotension can occur. The human body can produce approximately 8-12 liters of lymph during the day, but only a third of this production is driven and moved by muscle contraction; two-thirds of the lymph is driven by the contraction of lymphatic smooth muscle [40].

Diaphragm, venous movement, and lymph

DM is essential for creating central and peripheral pressures for optimal venous flow and outflow [20]. During inspiration, venous flow is facilitated into the thorax by creating negative intrathoracic pressure, while abdominal pressure is increased by "squeezing" the venous system. During DM contraction, the vena cava collapses, increasing abdominal pressure; these two factors impede venous return from the lower limbs, both at rest and under stress [41,42]. During expiration, venous return from the lower limbs to the hepatic venous system is favored [42].

During inspiration, the venous volume towards the right atrium increases, the pressure gradient of the two right cardiac chambers increases, and the elongation of the myocardial fibers increases (improving the preload) [43]. Inspiration improves the right cardiac stroke volume through the Frank-Starling mechanism; the left end-diastolic volume is also greater, as well as improving cardiac chronotropy [43]. The pressure difference generated by the DM allows the creation of the appropriate pressures for optimal cardiac output. During inspiration, venous outflow at the cerebral level is facilitated (the arterial influx increases with expiration); the blood volumes moved into/from the central nervous system are comparable to the volumes moved with cardiac work [44]. During inspiration, supradiaphragmatic venous flows are directed toward the heart-lung system, and venous fluids from the lower limbs are impeded in their movement toward the abdomen. This mechanism does not reflect the classic pumping mechanism.

DM activity stimulates the production of lymphatic fluids, while the presence of apnea or diaphragmatic dysfunction will prevent this formation [26]. DM is essential for the collection of lymph from the entire subdiaphragmatic area (abdomen, pelvis, and lower limbs), and part of the parietal pleural lymph. The periphery of the diaphragm is covered by mesothelial cells, which create innumerable stomata with unidirectional valves that open to collect the lymphatic flow towards predisposed spaces or lacunae [45]. The pleural cavity relates to the lymphatic system and the peritoneal cavity (they share a coelomic origin); a portion of the parietal lymph is directed towards the diaphragm, through the inferior ligaments of the lung (rich in lymphatic vessels) in close contact with DM [26,46]. DM is rich in lymphatic vessels, which empty their contents into the Chyli cistern (and then towards the thoracic duct); the pleural lymph collected by the diaphragm is pushed towards the peritracheobronchial lymph nodes [26,47]. Lymphatic movement is determined by several factors, including breathing.

If DM were just a pump, it wouldn't directly absorb lymph from various structures. Instead, it's designed to collect and distribute lymph.

DM is essential for the movement of neurofluids such as glymph and cerebrospinal fluid (CSF). Glymph is an ultrafiltrate of CSF and interstitial fluids. In the brain, approximately 10% of fluids are derived from blood, approximately 10% is CSF; the remainder comes from cellular fluids [48]. Glymph is essential for cleaning the nervous system, eliminating proteins such as amyloid-beta and tau, which, if in excess, can cause long-term neurological damage [44]. CSF has multiple functions, including acting as a mechanical shock absorber for nervous structures, allowing systemic homeostatic continuity, and as a crossroads of information for the central and peripheral nervous system, the lymphatic system, and the immune system [49]. Breathing mobilizes a greater amount of glymph/CSF than the heartbeat; the latter moves only about 15-25% of the total cranial fluids [50,51].

Glymphatic fluids are aided in their flow by vasomotion thanks to the predominant intervention of the parasympathetic system, while the synthesis of norepinephrine stimulates vasoconstriction [52]. Correct movement of DM stimulates the parasympathetic response, indirectly aiding glymphatic flow-outflow [6]. During respiratory acts, the brain mass and spinal cord undergo a cranial (with inspiration) and caudal (expiration) movement of 2-3 mm in healthy subjects, allowing further facilitation of the flow-outflow of glymphatic fluids [53]. With a forced inspiration, the glymphatic flow displacement mechanism becomes the most predominant [54]. The diaphragm becomes fundamental for maintaining brain health, avoiding the accumulation of toxic metabolites.

During inspiration, CSF at the spinal level is pushed cranially, while during expiration, it has a caudal direction, with an average movement of about 95.6 mm, with an average velocity of 0.60 to 1.59 mL/s for the cervical region, 0.46 to 3.17 mL/s at the thoracic level, 0.75 to 3.64 mL/s in the lumbar area [55,56]. The movement of neural fluids within the skull and spinal column is not linear, but chaotic and nonlinear movements (spinal subarachnoid space); they are moved without following the concept of a “pump”, but by different and non-uniform stimuli [57-59].

We remind you that further, more specific studies will be needed to measure the movement of neural fluids with breathing, in the presence of various factors, such as the influence of continuous positive airway pressure (CPAP), and concomitant pathologies.

Other non-pumping diaphragmatic functions

The diaphragmatic crural area acts as an "external sphincter" of the lower esophagus [43]. When the DM descends during inspiration, the Low's muscle and the transversus intertendinous muscle relax. During inspiration, the esophageal hiatus tends to narrow. With the relaxation of the two muscles innervated by the vagus nerve, the esophageal hiatus does not close completely, and in the presence of a bolus, passage to the stomach is assured [21]. During expiration, the esophageal hiatus tends to increase in diameter, while the two crural muscles contract: this balance between the phrenic and vagus nerves is fundamental for correct gastric function [21].

In the presence of inflammation and to facilitate the collection of extravasated interstitial fluid, the immune system activates a series of biochemical events capable of stimulating lymphangiogenesis and improving cellular cleansing by modulating the pathological environment [60,61]. In the presence of peritoneal inflammation, the diaphragm plays an important role, undergoing the mechanism of lymphangiogenesis and promoting the drainage of peritoneal fluid [60]. The presence of chronic stress induces a neuroimmune response towards an inflammatory environment, immunosuppression, with a more accentuated activity of the sympathetic system [16]. Adequate breathing can stimulate the parasympathetic system, influencing the immune system towards a more physiological environment, lowering pro-inflammatory cytokines and cortisol [16].

Correct diaphragmatic activation allows, through complex central and peripheral neurobiochemical mechanisms, the control of the emotional state and the cognitive sphere. Each inhalation activates brain areas that increase attention, reactions, concentration, and memory, as well as the emotional aspect is more controlled [62]. The cortical and subcortical networks that are activated most, and that allow the modulation of behavior, put in communication the olfactory bulb, the frontal and prefrontal cortex, the limbic area, the nucleus accumbens, the main and accessory motor cortical area, the somatosensory cortical area, and the nucleus of the solitary tract (NTS) [63]. 

**Figure 2:**
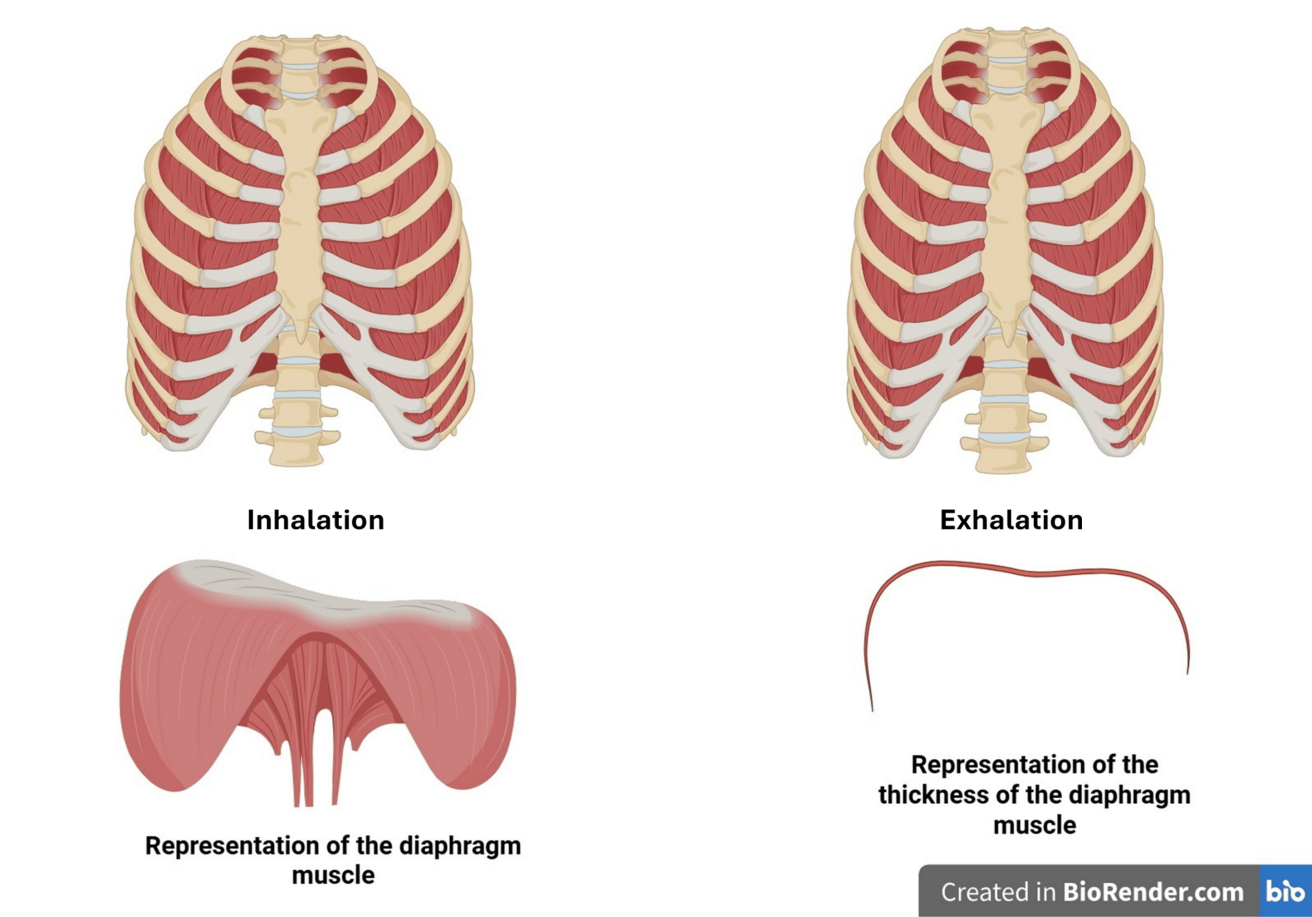
A schematic representation of how the rib cage moves during exhalation and inhalation (top right and left, respectively); a schematic representation of the morphology of the diaphragm muscle (bottom right) and its thickness (bottom left) The image is the property of Bordoni Bruno via a subscription to Biorender.com

DM influences neuromotor expression. Diaphragmatic activation activates most of the body's receptors (shifting fluid pressures), which, via the spinosolitary pathway to the NTS and the spinotrigeminal pathway, involve the cerebellar and vestibular areas; finally, the motor and limbic cortex networks will be activated, from which efferents will start to increase the activity of the parasympathetic system and a decline in the activity of the sympathetic system [62,64]. This network allows for better neuromotor activity, with greater expressed force. DM is so important for neuromotor expression that, when we are standing, to maintain posture and movement, a third of the diaphragm is already contracted. On the one hand, to maintain constant stimulation of the exposed neural networks, and on the other, to maintain the correct pressures of the dorsolumbar area (Figure 2) [62,64].

Correct and deep inspiration can raise the pain threshold and concentration, as well as maintain a physiological posture, thanks to the stimulation of the proprioceptive system and the relevant cortical areas, with an increase in parasympathetic activity [1,4,7,65-67]. A little-considered aspect is the anatomical pathway of the phrenic nerve. The phrenic nerve contains a percentage of catecholaminergic fibers; the nerve does not terminate in the diaphragm. It continues beyond DM, forming subdiaphragmatic phrenic ganglia, anastomosing with the celiac and aorticorenal ganglion, and terminating in the suprarenal gland. Furthermore, the phrenic nerve innervates the biliary area, Glisson's capsule, and the greater curvature of the stomach [11,12,20]. We don't know exactly why these neural connections exist, but we hypothesize that they play a role in visceral function and the stress response (hypothalamic-pituitary-adrenal axis).

DM is important for coughing, expectoration, and, most likely, for optimal control of the tongue complex to prevent obstructive sleep apnea (OSA) [4,31,68]. A functional DM is important for pelvic floor function, influencing muscle activation or inhibition. When DM contracts, the pelvic muscles relax, creating space for diaphragmatic descent; if this central and peripheral neural mechanism fails, pelvic and visceral problems may arise, such as nonspecific pain and pelvic visceral functional alterations [69,70].

The clinical perspective

If we consider DM to be flattened in chronic respiratory diseases, this contradicts studies demonstrating that diaphragmatic function improves after rehabilitation, as do respiratory parameters [71,72]. Indeed, as DM movement improves, its morphology should change. In reality, in the current scientific literature and to the authors' knowledge, there is no study demonstrating that DM is no longer flattened. As previously stated in this article, DM does not change its morphology. The morphological parameter used to assess whether DM is functional or nonfunctional is not useful for clinical change. It would probably be more appropriate to evaluate ZOA.

**Figure 3:**
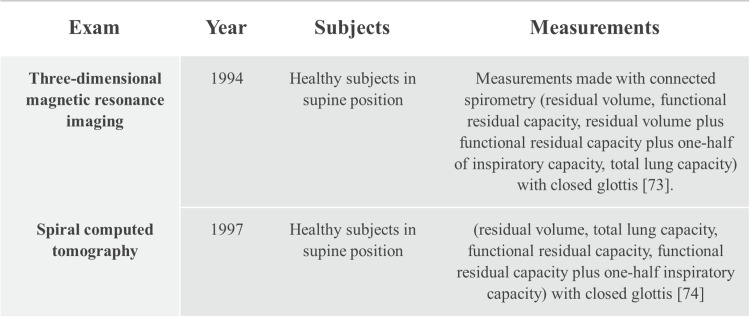
Summary of decades of knowledge about the fact that the diaphragm must be considered in its three-dimensionality and that its morphology does not change during breathing

In a human model with nuclear magnetic images, on the sagittal plane, it is demonstrated that DM does not contract like a pump or a piston [73,74]. DM generates non-homogeneous pressures, both due to the localization of different viscera and to its conformation; this means that it does not produce a pump effect [74]. ZOA is larger on the right side by approximately 21% compared to the left side; its surface area changes depending on the posture, where in the upright position it will have a surface of approximately 900 square cm and in the supine position of approximately 1,151 square cm [74]. The length of the fibers in proximity to ZOA is approximately 9 cm on the right, while they will be approximately 7 cm on the left. When DM contracts, it is the surface of the lung that fills ZOA. The morphology of the diaphragmatic domes does not vary; it is the morphology of the lung that varies according to the movement of ZOA (Figure 3) [74].

When DM lowers, its diameter widens (antero-posterior and lateral), but the shape of the diaphragmatic domes does not change, both in healthy subjects and in patients with chronic obstructive pulmonary disease [74-76]. The movement it expresses most during inspiration is oblique, not caudal [22]. The position of the body can influence the geometry of DM. In the supine position, the curve of the diaphragmatic dome is more evident compared to an upright position (orthostatism); overall, the shape of the domes does not vary significantly [77]. To understand how DM works clinically, it is necessary to have a three-dimensional vision; it is more useful to evaluate ZOA and the difference in movement between inspiration and expiration [34,77].

A recent study has shown that measuring DM thickness is not necessarily correlated with a decrease in contractile tissue (sarcopenia) [78]. Most likely, according to current literature, the clinical instrumental pathway for the evaluation and a more comprehensive functional assessment of DM should use a three-dimensional strategy. Two-dimensional instrumental tests (such as ultrasound, which is rapid and inexpensive) can help assess some clinical variables in critically ill patients, such as excursion, to decide whether the patient can be extubated, or how the patient moves after surgery [79,80].

How can we define the diaphragm muscle with a new terminology that allows us to appreciate its functional complexity, as it is completely different from other skeletal muscles?

Connector muscle

The diaphragm muscle connects multiple bodily functions, and these connections are crucial for physical health, postural behavior, and cognitive and emotional quality. Considering its neurological connections (phrenic and vagus nerves, and anastomoses with some sympathetic ganglia), the diaphragm goes beyond the mere function of a skeletal muscle. The term "pump" or "pressure generator" for moving air, or the term "flow modulator" for venous/lymphatic/neurofluids, are inaccurate in describing how air and neurofluids move. Air moves with vortex movements and at different speeds, while neurofluids move with nonlinear movements and at non-homogeneous speeds [81-85].

The diaphragm is a muscle with the ability to connect body systems, a systemic connector.

To the authors' knowledge, no muscle in the body has been associated with the concept of a connector with respect to the functions expressed by the same muscle. We propose this terminology to indicate the diaphragm's multiple functions, but we are open to further improvements if other clinicians and researchers wish to explore a more appropriate etymological framework.

The idea of considering DM as a mere "pump" should be eliminated from scientific jargon.

## Conclusions

The article proposes the term "connector muscle" to indicate the diaphragmatic function, rather than the usual term "pump," highlighting the myriad complex functions of DM. The term "pump" does not reflect current knowledge regarding the systemic influence of diaphragmatic movement. Furthermore, the article reiterates that during contraction or inspiration, the morphology of the diaphragmatic domes does not change, and overall, it does not "flatten." We urge other researchers and authors to endorse this adjective (connector) to generally define DM, or to suggest another etymology that better captures the diaphragmatic functions. It is useful to remember that, in a clinical setting, it is impossible to understand or evaluate DM without considering the multiple actions it performs; that is, we must remember that it is not simply a respiratory "pump" muscle.
